# Influence of insufflated carbon dioxide on abdominal temperature compared to oesophageal temperature during laparoscopic surgery

**DOI:** 10.1007/s00464-020-08196-x

**Published:** 2020-12-01

**Authors:** Philipp Groene, Ufuk Gündogar, Klaus Hofmann-Kiefer, Roland Ladurner

**Affiliations:** 1 Department of Anaesthesiology, University Hospital, LMU Munich, Marchioninistraße 15, 81377 Munich, Germany; 2 Department of General, Visceral and Transplantation Surgery, University Hospital, LMU Munich, Munich, Germany

**Keywords:** Temperature, Laparoscopic, Hypothermia, Carbon dioxide

## Abstract

**Background:**

Body core temperature is an important vital parameter during surgery and anaesthesia. It is influenced by several patient-related and surgery-related factors. Laparoscopy is considered beneficial in terms of a variety of parameters, for example, postoperative pain and length of hospital stay. Non-humidified, non-warmed insufflated CO_2_ applied during laparoscopy is standard of care. This prospective observational trial therefore evaluates the impact of non-humidified CO_2_ at room temperature on abdominal temperature and its correlation to body core temperature.

**Methods:**

Seventy patients undergoing laparoscopic surgery were included in this prospective observational study. Temperature was measured oesophageal and abdominal before induction of anaesthesia (T1), right before skin incision (T2), 15 min, 30 min and 60 min after skin incision. All patients were treated according to actual guidelines for perioperative temperature measurement.

**Results:**

Body core temperature and abdominal temperature correlated moderately (*r* = 0.6123; *p* < 0.0001). Bland–Altman plot for comparison of methods showed an average difference of 0.4 °C (bias − 0.3955; 95% agreement of bias from − 2.365 to 1.574). Abdominal temperature further decreased after establishing pneumoperitoneum (T2: 36.2 °C (35.9/36.4) to T5: 36.1 °C (35.6/36.4); *p* < 0.0001), whereas oesophageal temperature increased (T2: 36.2 °C (35.9/36.4) to 36.4 °C (36.0/36.7); *p* = 0.0296). Values of oesophageal and abdominal measurement points differed at T4 (36.3 °C (36.0/36.6) vs. 36.1 °C (35.4/36.6); *p* < 0.0001) and T5 (36.4 °C (36.0/36.7) vs. 36.1 °C (35.6/36.4) *p* = 0.0003).

**Conclusion:**

This prospective observational trial shows the influence of insufflated, non-humidified carbon dioxide at room temperature on abdominal temperature during laparoscopic surgery. We show that carbon dioxide applied at these conditions decreases abdominal temperature and therefore might be a risk factor for perioperative hypothermia.

Body core temperature is an important vital parameter during surgery and anaesthesia. Body core temperature influences several biochemical processes and physiological pathways. A Europe-wide survey in 2007 showed that in only 19% of surgeries under general anaesthesia, body temperature was documented [1]. However, perioperative temperature management is an important part of perioperative management.

A body core temperature below 36 °C is normally called “hypothermia”. Perioperative temperatures below this value reduce patients’ outcome due to negative effects on various physiological functions. Cardiovascular events like myocardial infarction, acute coronary syndrome (ACS) or arrhythmias increase significantly [2–4]. Likewise, blood coagulation is impaired, which leads to increased intraoperative blood losses and, as a consequence, increased transfusion requirements [4–6]. Perioperative hypothermia also impacts tissue oxygenation and leads to vasoconstriction resulting in an increased frequency of wound infections [7–9]. Last but not least, hypothermic patients are at discomfort in the recovery room. Hypothermia-related shivering increases oxygen consumption and as a consequence increases the risk of adverse cardiovascular events [10–12].

Body temperature during surgery is influenced by several factors which can be subdivided into different categories such as anaesthesia-related, surgery-related, environmental, or patient-related risk factors. Surgery-related risks include the surgical technique, the duration of surgery, the extent of the procedure, and the amount of irrigation fluid used [13]. Some studies showed that laparoscopic surgery can negatively influence body temperature by using carbon dioxide (CO_2_) at room temperature [14–16]. Nevertheless, laparoscopic surgery mostly is performed without pre-warming and humidifying CO_2_.

Therefore, the aim of this prospective, observational study was to investigate the influence of insufflated, non-humidified CO_2_ at room temperature on abdominal temperature compared to oesophageal temperature over time. Primary outcome was the correlation between both measurements.

## Material and methods

The study was approved by the LMU Munich ethics committee (No. 17-143) and performed in accordance with the Declaration of Helsinki. This study also follows the CONSORT guidelines. Written informed consent was obtained from all patients participating in this prospective, observational study.

Inclusion criteria were planned laparoscopic surgery, age > 18 and patient’s written, informed consent. Exclusion criteria were age < 18, change to open surgery, pregnancy, combined epidural–general anaesthesia and patient’s denial.

Right before induction of anaesthesia, the first temperature measurement was made (T1). One min before surgical incision/establishing pneumoperitoneum the next temperature measurement was carried out (T2). Further temperature measurements took place at 15, 30 and 60 min after surgical incision (T3 and T5). Temperature was measured sublingually (Digitemp, servoprax GmbH: Wesel, Germany) before induction of anaesthesia (T1) and oesophageal from then on (T2-T5) according to recommended methods of temperature measurement [17]. Intra-abdominal measurement was done with a urinary catheter with integrated temperature measurement which was inserted through the camera trocar (T3-T5). It was placed right below the omentum and between the intestinal loops. Temperature was measured continuously except for the first measurement which was measured sublingually once.

Pre-warming of the patients was started immediately after arriving in the operating theatre using a whole-body blanket and warm air (Bair Hugger, 3 M, Maplewood, USA) (38 °C according to the manufacturer’s instructions). The patients were continuously and actively heated as required by the German guidelines during surgery [13, 17]. During surgery, patients received a preheated blanket over their legs and an actively warming blanket across thorax and upper extremities. Temperature in the operating theatre was set to 21 °C as recommended in the guideline and controlled by the anaesthetist.

### Statistics

Sample size calculation was done with G*Power version 3.1 (HHU Düsseldorf, Germany) and based on an expected great effect of carbon dioxide (effect size dz = 0.3) at room temperature on abdominal temperature. A sample size of 70 patients was estimated to provide a power of 80% for detecting a statistically significant difference at a level of 0.05 (z-test; inequality of two dependent Pearson r’s).

## Results

We included 70 patients undergoing laparoscopic surgery. Performed surgeries included appendectomy (*n* = 10), cholecystectomy (*n* = 20), adrenalectomy (*n* = 9), transabdominal preperitoneal hernia repair (TAPP; *n* = 8), sigmoid colectomy (*n* = 13), incisional hernia repair (*n* = 5), iliacal adenectomy (*n* = 1), adhesiolysis (*n* = 1), hepatic resection (liver cyst) (*n* = 1), implantation of abdominal dialysis catheter (*n* = 1) and hiatus hernia repair (*n* = 1). Detailed patient’s characteristics are displayed in Table 1.

**Table 1:** Patient characteristics (*n* = 70). Data presented as mean ± standard deviation

Gender: women/men (*n*; %)	34/36 (49/51)
Age (years)	54 ± 16
BMI (kg m^−2^)	26.9 ± 6.0
ASA status (*n*; %)	
1	5 (7)
2	41 (59)
3	23 (33)
4	1 (1)
Time from induction to skin incision (min)	43 ± 15
Time of surgery (min)	77 ± 46
Blood loss (ml)	45 ± 60
Crystalloid (ml)	1150 ± 600
Carbon dioxide insufflated (ltrs)	147 ± 145

*ASA* American society of Anaesthesiologists; *BMI* body mass index

Median blood loss and infused crystalloid were 20 ml (0/50) and 1000 ml (800/1500), respectively. None of the patients received colloids or packed red blood cells. All patients had general anaesthesia without epidural anaesthesia.

**Fig. 1:**
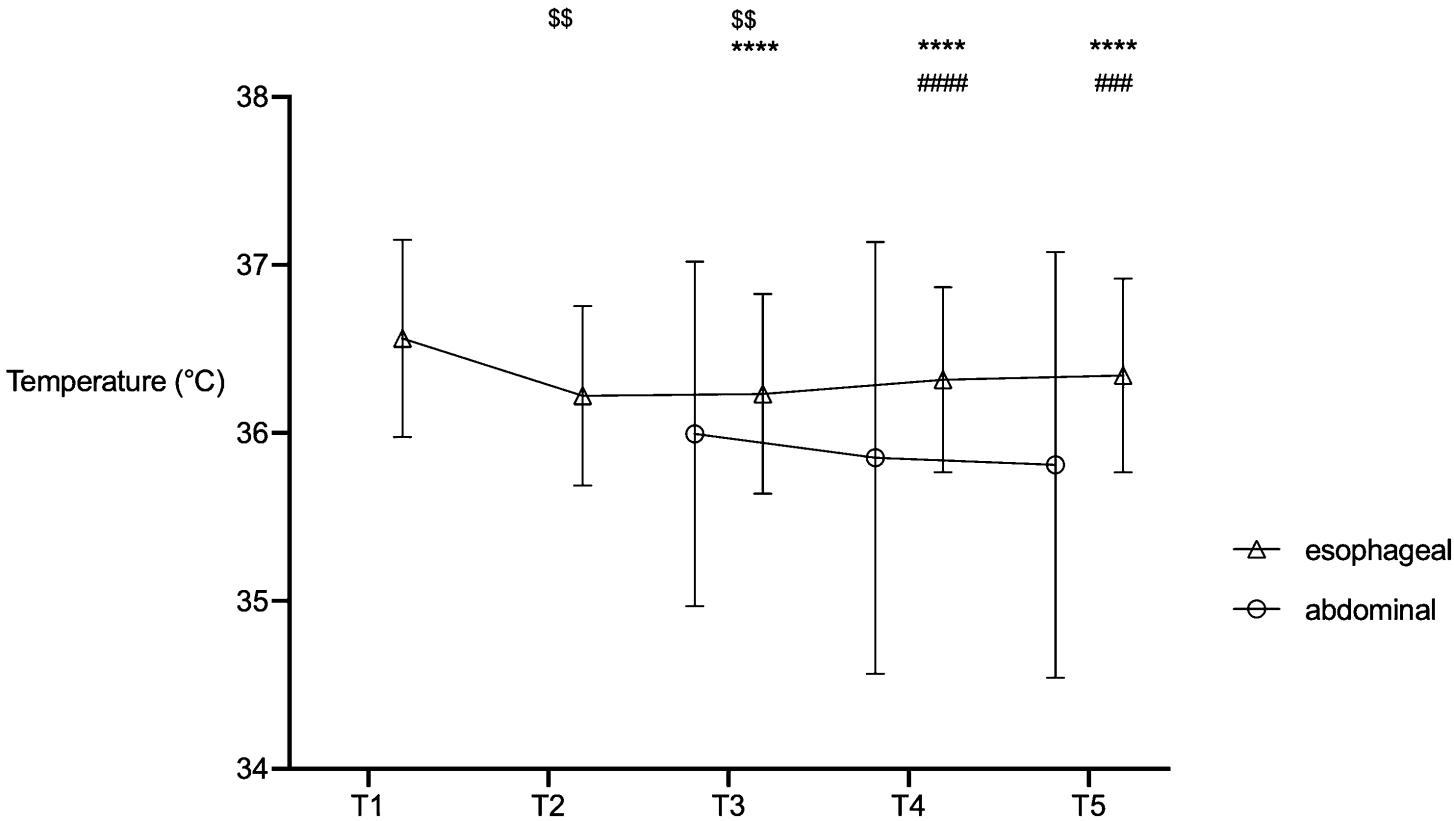
Temperature over time course. **T1:** before anaesthesia induction (sublingual measurement); **T2:** 1 min before skin incision (establishing pneumoperitoneum; easophageal measurement); **T3-5:** 15, 30, 60 min after skin incision (oesophageal and abdominal measurements); $ vs. “anaesthesia induction” (oesophageal measurement). *vs. “anaesthesia induction” (abdominal measurement). ^#^Abdominal vs. oesophageal measurement. *p < 0.05; **p < 0.01; ***p < 0.001; ****p < 0.0001

Before induction of anaesthesia, median temperature was 36.6 °C (36.2/36.9) and decreased despite active warming to 36.2 °C (35.9/36.4) right before incision (establishing pneumoperitoneum) (Fig. 1; *p* = 0.005). Temperature values remained below the initial temperature (T1) until 15 min after incision (36.2 °C (35.9/36.5); *p* = 0.006) for oesophageal temperature measurement (Fig. 1). After that, temperature did not differ compared to T1. Temperature measured abdominally remained below initial temperature values until 60 min after skin incision (pneumoperitoneum) (T3: 36.2 °C (35.5/36.6); T4: 36.1 °C (35.4/36.6); T5: 36.1 °C (35.6/36.4); all *p* < 0.0001). Compared to temperature right before pneumoperitoneum abdominal temperature further decreased (T2: 36.2 °C (35.9/36.4) to T5: 36.1 °C (35.6/36.4); *p* < 0.0001) whereas oesophageal temperature increased (T2: 36.2 °C (35.9/36.4) to 36.4 °C (36.0/36.7); *p* = 0.0296). Values of oesophageal and abdominal measurement points differed at T4 (36.3 °C (36.0/36.6) vs. 36.1 °C (35.4/36.6); *p* < 0.0001) and T5 (36.4 °C (36.0/36.7) vs. 36.1 °C (35.6/36.4) *p* = 0.0003).

We observed a moderate correlation (*r* = 0.6123, *p* < 0.0001) between intra-abdominal and oesophageal temperatures at all times of measurement. Additionally, we performed a Bland–Altman plot to compare both methods, which showed an average difference of 0.4 °C (bias − 0.3955; 95% agreement of bias from − 2.365 to 1.574).

## Discussion

This prospective observational trial shows the influence of insufflated, non-humidified carbon dioxide at room temperature and on abdominal temperature during laparoscopic surgery. We were able to show that carbon dioxide applied under these conditions lessens abdominal temperature and therefore can be a risk factor for perioperative hypothermia. Correlation between oesophageal and abdominal temperature was moderate.

In the last decade, various risk factors for the development of perioperative hypothermia have been identified [13]. Both, patient-independent and patient-related factors influence the risk of hypothermia. Factors related to the patient are age, pre-existing low body temperature and patients with diabetic neuropathy [13]. Surgery-related factors include the duration and extent of surgery [13]. The influence of open versus laparoscopic surgery on body temperature is still not clear. Two randomized controlled studies investigated this aspect in the last years [18, 19]. Meta-analysis of these two studies favoured neither of both [13]. There was no significant difference intraoperatively concerning body temperature, but Nguyen et al. reported higher body temperatures at the post-anaesthesia care unit after laparoscopic and open gastric bypass (PACU) [19, 20]. However, considering the physiology of open surgery, it should have proven to be disadvantageous. The longer duration of surgery with laparoscopic technique was considered to be one reason for the equality results. Another reason which came into focus was CO_2_ used during laparoscopy. Normally it is used not humidified and at room temperature (20–22 °C). Some groups evaluated the effects of insufflated CO_2_ on intraoperative body temperature as well as on postoperative pain and length of hospital stay [15, 21]. The two meta-analyses of Dean et al. and Balayssac et al. were able to show positive effects of warmed, humidified CO_2_ compared to controls without warming [16, 21]. Jiang et al., however, demonstrated that warmed, humidified CO_2_ or combined forced air warming with CO_2_ at room temperature are equivalent [15].

In this context, abdominal temperature was measured in two studies and compared to the body core temperature, mostly measured in the oesophagus [19, 20]. In a small study (*n* = 20), Saad et al. evaluated intra-abdominal temperature and oesophageal temperature in groups with and without warmed CO_2_ but did not compare them. The study only compared groups concerning type of insufflation [20]. Nguyen et al. compared open and laparoscopic surgery in 101 patients, but only in 30 patients undergoing laparoscopic surgery intra-abdominal temperature was measured. Nevertheless, the group showed a significant difference between body core temperature (oesophagus) and abdominal temperature [19].

The data of our study support the results of Nguyen et al., but included a higher number of patients and external body warming was practiced according to the actual guidelines for perioperative temperature management [13, 17]. All patients were actively warmed by forced air warming pre-operatively and intraoperatively. Still, CO_2_ applied at room temperature significantly cooled down the abdomen. Although there already is evidence favouring warmed CO_2_ based on data measuring body core temperature, we wanted to demonstrate the negative effect of cool CO_2_ on abdominal temperature, which previously was shown only in studies with low numbers of patients. Hence, this study supports the actual results of Dean et al. and Balayssac et al., favouring warmed, humidified CO_2_ [16, 21].

## Limitations

Our study has some limitations. First, different types of laparoscopic surgery were included in the study which may have had a (small) impact on the comparability the results. Second, the position of the oesophageal catheter was only verified by looking into the patients’ mouth and by groping. Thus, there might have been small differences concerning the exact intra-oesophageal position of the catheter between patients, which might have led to small deviations concerning temperature measurements. Fluid management (e.g. total volume and temperature of the volume) could have influenced the measurement. Nevertheless, all fluids were at room temperature and it was the same for all patients.
